# Head-to-head comparison of composite and individual biomarkers to predict clinical benefit to PD-1 blockade in non-small cell lung cancer

**DOI:** 10.1371/journal.pone.0293707

**Published:** 2024-07-31

**Authors:** Karlijn Hummelink, Vincent van der Noort, Mirte Muller, Robert D. Schouten, Michel M. van den Heuvel, Daniela S. Thommen, Egbert F. Smit, Gerrit A. Meijer, Kim Monkhorst

**Affiliations:** 1 Department of Pathology, Division of Diagnostic Oncology, Netherlands Cancer Institute, Amsterdam, The Netherlands; 2 Department of Thoracic Oncology, Division of Medical Oncology, Netherlands Cancer Institute, Amsterdam, The Netherlands; 3 Department of Biometrics, Netherlands Cancer Institute, Amsterdam, The Netherlands; 4 Division of Molecular Oncology and Immunology, Netherlands Cancer Institute, Amsterdam, The Netherlands; Taichung Veterans General Hospital, TAIWAN

## Abstract

**Background:**

The efficacy of PD-1 blocking agents in advanced NSCLC has shown prolonged effectiveness, but only in a minority of patients. Multiple biomarkers have been explored to predict treatment benefit, yet their combined performance remains inadequately examined. In this study, we assessed the combined predictive performance of multiple biomarkers in NSCLC patients treated with nivolumab.

**Methods:**

Pretreatment samples from 135 patients receiving nivolumab were used to evaluate the predictive performance of CD8 tumor-infiltrating lymphocytes (TILs), intratumoral (IT) localization of CD8 TILs, PD-1 high expressing TILs (PD1^T^ TILs), CD3 TILs, CD20 B-cells, tertiary lymphoid structures (TLS), PD-L1 tumor proportion score (TPS) and the Tumor Inflammation score (TIS). Patients were randomly assigned to a training (n = 55) and validation cohort (n = 80). The primary outcome measure was Disease Control at 6 months (DC 6m) and the secondary outcome measure was DC at 12 months (DC 12m).

**Results:**

In the validation cohort, the two best performing composite biomarkers (i.e. CD8+IT-CD8 and CD3+IT-CD8) demonstrated similar or lower sensitivity (64% and 83%) and NPV (76% and 85%) compared to individual biomarkers PD-1^T^ TILs and TIS (sensitivity: 72% and 83%, NPV: 86% and 84%) for DC 6m, respectively. Additionally, at 12 months, both selected composite biomarkers (CD8+IT-CD8 and CD8+TIS) demonstrated inferior predictive performance compared to PD-1^T^ TILs and TIS alone. PD-1^T^ TILs and TIS showed high sensitivity (86% and 100%) and NPV (95% and 100%) for DC 12m. PD-1^T^ TILs could more accurately discriminate patients with no long-term benefit, as specificity was substantially higher compared to TIS (74% versus 39%).

**Conclusion:**

Composite biomarkers did not show improved predictive performance compared to PD-1^T^ TILs and TIS alone for both the 6- and 12-month endpoints. PD-1^T^ TILs and TIS identified patients with DC 12m with high sensitivity. Patients with no long-term benefit to PD-1 blockade were most accurately identified by PD-1^T^ TILs.

## Introduction

The success of monoclonal antibodies targeting the inhibitory receptor programmed cell death protein 1 (PD-1) and its ligand programmed death-ligand 1 (PD-L1) has changed the therapeutic landscape of advanced stage non-small cell lung cancer (NSCLC). A subset of patients treated with these PD-1/PD-L1 blocking agents experience durable responses, translating into a significant survival advantage [1–7]. However, the majority fails to derive durable clinical benefit, underscoring the need for predictive biomarkers to support treatment decision-making in clinical practice. Specifically, the identification of biomarkers capable of excluding patients unlikely to benefit from PD-1/PD-L1 blockade therapy can prevent unnecessary side effects and contribute to the reduction of health care costs.

The assessment of tumor PD-L1 expression through immunohistochemistry (IHC) has been a focal point in numerous clinical trials as a potential predictive biomarker [8]. Although a positive correlation between PD-L1 expression and treatment outcomes has been observed in advanced stage NSCLC patients [1, 5–7], a considerable proportion (60% to 70%) of patients with PD-L1 positive tumors do not respond [1, 2, 5]. Besides this, PD-L1 assessment by IHC is hampered by intratumor heterogeneity, interassay- and interobserver variability as well as pre-analytical variation [9–14]. Tumor Mutation Burden (TMB), reflecting the number of somatic mutations as a surrogate for potential tumor antigenicity, has also shown predictive potential. However, its clinical implementation faces challenges, including the lack of a robust and predictive TMB cut-off and technical issues related to variation across platforms [15–17].

Given these challenges, there is an urgent need for biomarkers that can more accurately predict responses to PD-1/PD-L1 blockade in advanced NSCLC. Since this treatment regimen is thought to reinvigorate tumor-reactive T cells [18–20], several T cell markers have been investigated. For example, the density of CD8^+^ tumor infiltrating lymphocytes (TILs) has been correlated with responses to PD-1 blockade in various cancer types, including melanoma [18], colorectal cancer [21], and NSCLC [22, 23]. In addition, previous work showed that a distinct T cell population, termed PD-1^T^ TILs, can predict clinical benefit in NSCLC [24, 25]. These PD-1^T^ TILs predominantly localize in tertiary lymphoid structures (TLS) [24, 25]. Notably, B cells, critical components of these TLS, have also been associated with response to PD-1 blocking agents [26–28]. Other studies have developed predictive RNA expression signatures, such as the “tumor inflammation signature” (TIS), characterizing features of immune activity in the tumor microenvironment (TME) [29–31].

While these individual biomarkers show predictive potential, their accuracy is limited, likely due to the multifaceted nature of the antitumor immune response. Therefore, combining biomarkers holds promise for enhancing predictive accuracy, as demonstrated previously for combinations like TMB with PD-L1 [32, 33] and CD8 TILs with PD-L1 [22, 34]. Thus, the primary objective of this study is to investigate the performance of biomarker pairs, including CD8, PD-1^T^ and CD3 TILs, CD20^+^ B cells, TLS, PD-L1, and TIS, and compare their efficacy against individual biomarkers in predicting clinical benefit to PD-1 blockade in NSCLC.

## Methods

### Patients, endpoints and samples

In this study, 162 patients with pathologically confirmed stage IV NSCLC were eligible for efficacy analysis. All enrolled patients started second or later line monotherapy nivolumab, administered intravenously at a dose of 3mg/kg every two weeks for at least one dose, between October 2014 and August 2017 at the Netherlands Cancer Institute/Antoni van Leeuwenhoek hospital (NKI-AVL), the Netherlands. Patients with tumors harboring known sensitizing EGFR mutations or ALK translocations were excluded from treatment. A randomization process was employed to allocate patients into a training and validation cohort. This randomization was stratified by treatment outcome at 6 months and at 12 months. Since we could only generate gene expression data in 68/162 (42%) of patients’ tumors, additional stratification was done by whether mRNA expression analysis was performed or not. Stratification for missing values of other biomarkers was not performed, as the number of excluded samples per biomarker remained relatively low, ranging from 1 to 32 (see S1 Fig and later in this section).

Response to treatment was evaluated according to the Response Evaluation Criteria in Solid Tumors (RECIST) version 1.1. Patients with progressive disease (PD) who were not evaluable for response assessment were designated by the treating physician as having PD. The primary clinical outcome was Disease Control (DC), defined as achieving a complete response (CR), partial response (PR) or maintaining stable disease (SD) at the 6-month mark following the initiation of treatment. As a secondary outcome measure, DC 12m was employed, representing the persistence of CR, PR or SD for a duration of 12 months or more. This secondary endpoint aimed to serve as an indicator of the long-term efficacy to PD-1 blockade therapy.

Pretreatment formalin-fixed paraffin embedded (FFPE) tumor tissue samples were collected from all patients. Written informed consent for the research usage of material, not essential for diagnostic purposes, was obtained from each patient by an institutionally implemented opt-out procedure. The study was conducted in accordance with the Declaration of Helsinki. The data was accessed for research purposes after the approval by the Institutional Review Board (IRB) of the Netherlands Cancer Institute on January 11, 2018 (CFMPB586). After K.H., M.M., R.D.S., M.M.H., E.F.S. and K.M. retrieved archived tumor samples and response data from medical records, all patients were pseudonymized. PD-1^T^ TIL and PD-L1 tumor proportion score (TPS) data for 94 samples as well as tertiary lymphoid structures (TLS) and CD20^+^ B cell data for 91 samples were used from previous work [25]. In 27 patients, none of the biomarkers could be assessed because samples did not contain tumor tissue. In one sample no tumor tissue was left for CD8 and PD-1^T^ TIL analysis, and five samples had insufficient tumor tissue for CD3 TIL, TLS and CD20^+^ B cell analysis (S1 Fig). An additional number of 32 patients were excluded for PD-1^T^ TIL analysis based on the following criteria: samples contained less than 10,000 cells (n = 12), were obtained from endobronchial lesions (n = 16), contained abundant normal lymphoid tissue (n = 1) and showed fixation and/or staining artefacts (n = 2) (S1 Fig). As described before, we excluded bronchial biopsies because they frequently showed unspecific antibody staining due to mechanical damage, and lymph node resections due to presence of PD-1^+^ T cells in normal abundant lymphoid tissue, which could potentially lead to false positive results [25]. One sample was excluded for CD8 TIL, CD3 TIL, TLS, CD20^+^ B cell and PD-L1 analysis because of fixation/staining artefacts. One sample contained less than 2,000 cells and was excluded for CD8 TIL, CD3 TIL, TLS and CD20^+^ B cell analysis. A total of 67 patients (41%) were excluded for mRNA expression analysis due to low RNA yield and/or low RNA quality (S1 Fig).

### Immunohistochemistry

The CD8 immunostaining of samples was executed using the BenchMark Ultra autostainer Instrument (Ventana Medical Systems) on 3 μm paraffin sections from FFPE blocks. Initially, sections were baked at 75°C for 28 minutes and deparaffinised in the instrument with EZ prep solution (Ventana Medical Systems). Heat-induced antigen retrieval was carried out using Cell Conditioning 1 (CC1, Ventana Medical Systems) for 32 minutes. CD8 was detected using clone C8/144B (1/200 dilution, 32 minutes at 37°C, Agilent/DAKO). Bound antibody was detected using the OptiView DAB Detection Kit (Ventana Medical Systems). Slides were counterstained with Hematoxylin and Bluing Reagent (Ventana Medical Systems).

Immunostaining for PD-1 was carried out using clone NAT105 (Roche Diagnostics), for PD-L1 using clone 22C3 (Agilent/DAKO), and for CD68 using clone KP1 (Agilent/DAKO). For the double staining of CD20 (yellow) followed by CD3 (purple), clone L26 (Agilent/DAKO) (CD20) and clone SP7 (Thermo Fisher) (CD3) were used. All immunostainings were performed as described previously [25].

The immunostained slides for CD8, PD-1, PD-L1 and CD68 were subjected to scanning at a magnification of x20 with a resolution of 0.50 per μm^2^ using an Aperio slide AT2 scanner (Leica Biosystems). Immunostained slides for CD20-CD3 were scanned at x20 magnification with a resolution of 0.24 per μm^2^ using a 3Dhistech P1000 scanner. For manual scoring, PD-L1 and CD68 IHC images were uploaded onto Slide Score, a web platform designed for the manual scoring of digital slides using a scoring sheet (www.slidescore.com). CD8, PD-1^T^, CD3 TILs, CD20^+^ B cells and TLS were digitally scored as described below.

### Digital quantification of CD8 and PD-1^T^ TILs

Digital image analysis was performed by a trained MD (K.H.) and supervised by an experienced pathologist (K.M.) using the Multiplex IHC v1.2 module from the HALO^™^ image analysis software, version 2.3.2089.69 (Indica Labs). Researchers were blinded for clinical outcome. For the classification of CD8 lymphocytes in single stains, a computationally derived cut-off of 0.3 optical density (OD) was used, reflecting the intensity of the staining. This cut-off was established by manually optimizing the detection of CD8 positive stained cells in FFPE samples. An image analysis algorithm utilizing a 0.3 OD cut-off was generated for automated analyses of CD8 lymphocytes in subsequent FFPE samples. The quantification of PD-1^T^ TILs followed a previously described methodology [25].

The frequency of CD8 and PD-1^T^ TILs were determined as the number per mm^2^ tumor area. Tumor areas were digitally annotated as described previously [25]. PD-1^T^ TIL data from 94 samples were used from previous work [25] (S1 Table). For regional analysis of CD8 lymphocytes, classifiers were trained to distinguish stromal and tumoral regions, allowing for the separate quantification of CD8 lymphocytes in these distinct regions. The percentage of CD8 lymphocytes within tumoral regions (i.e. intra-tumoral (IT)) relative to total CD8 TILs was subsequently calculated (S1 Table).

### Scoring of tertiary lymphoid structures

The quantification of TLS and the combined number of TLS and lymphoid aggregates (TLS+LA) per mm^2^ tumor area was performed using the HALO^™^ image analysis software, version 2.3.2089.69 (Indica Labs). This analysis was conducted on a CD20-CD3 double immunostaining, following a previously established methodology [25]. TLS and TLS+LA data from 91 samples were used from previous work [25] (S1 Table).

### CD20 and CD3 quantification by digital image analysis

Digital quantification of CD20 and CD3 expression involved the measurement of the total area with CD20 and CD3 expression, relatively. This was performed using a pre-established image analysis algorithm from the Area Quantification version 1.0 module of HALO^™^ image analysis software (Indica Labs) [25]. The resulting CD20-positive and CD3-positive areas were normalized per mm^2^ tumor area. Cell numbers were not quantified as no reliable algorithm could be established due to dense clustering of CD20^+^ or CD3^+^ cells within and at the border of TLS. Tumor areas were digitally annotated as described previously [25]. CD20 data from 91 samples were used from previous work [25] (S1 Table).

### PD-L1 scoring

PD-L1 TPS was determined using the qualitative, clinical grade, laboratory developed, IHC assay (22C3 Agilent/DAKO) as described previously [25]. PD-L1 TPS data from 94 samples were used from previous work [25] (S1 Table). The CD68 staining was compared to the PD-L1 staining to exclude macrophages that express both CD68 and PD-L1, as their presence could potentially introduce false-positive results.

### RNA extraction and hybridization to nCounter tagset

The extraction of RNA from pretreatment FFPE samples and subsequent Nanostring analysis were performed as described previously [35].

### Statistical analysis

Patient characteristics were descriptively reported using mean ± SD, interquartile range (IQR) or frequencies (percentages). The Mann-Whitney test for continuous data, Fisher’s exact test for categorical data and linear-by-linear association test for ordinal data were used to assess differences in patient characteristics between cohorts (training and validation) and between outcome groups (DC vs PD). Statistical significance was considered at *P<0.05, **P<0.01, ***P<0.001 or ****P<0.0001.

Genes in the Tumor Inflammation Signature (TIS) are normalized using a ratio of the expression value to the geometric mean of the housekeeper genes specific to the TIS signature and then followed by log2 transformation. The TIS score, a weighted linear combination of the 18 gene expression values, was calculated as part of Nanostring’s intellectual property [29, 36] (S1 Table).

In the training cohort, univariate and bivariate logistic models were constructed for DC 6m and DC 12m using CD8 TILs, IT-CD8 T cells, PD-1^T^ TILs, CD3 TILs, TLS, TLS+LA, CD20^+^ B cells, PD-L1 and TIS. The bivariate models included an interaction term. The bivariate logistic model produces for each patient a number between 0 and 1, reflecting the probability (according to the model) of patients reaching DC 6m or DC 12m. Discriminatory ability was evaluated using the area under the receiver operating characteristic (ROC) curve. Predictive performance metrics (sensitivity, specificity, positive predictive value (PPV) and negative predictive value (NPV)) for different individual and composite biomarkers were calculated and comparisons were made using the McNemar test. A point on the ROC curve corresponding to 90% sensitivity for DC 6m or DC 12m was selected to determine specificity, NPV and PPV. The aim was to achieve an NPV of ≥90% and a specificity of ≥50%.

Two (closely related) non-parametric approaches were considered to obtain 90% sensitivity for predicting DC 6m and DC 12m from two biomarkers. In both methods, a cut-point was chosen for each of the two biomarkers and a patient was predicted positive (i.e. likely to respond to PD-1 blockade) if at least one (first method) or both (second method) biomarker values were above their respective cut-point values. The specificities obtained with these non-parametric methods were either equal to or worse than those obtained by the parametric method described above (i.e. via logistic regression). Therefore, these non-parametric methods were not used in this study.

Four training models were selected based on a cut-off that demonstrated the highest specificity and NPV at the predefined sensitivities for predicting DC 6m and DC 12m. This cut-off was then used to determine sensitivity, specificity, NPV and PPV in the validation cohort.

## Results

### Biomarker characteristics and demographics

**Fig 1:**
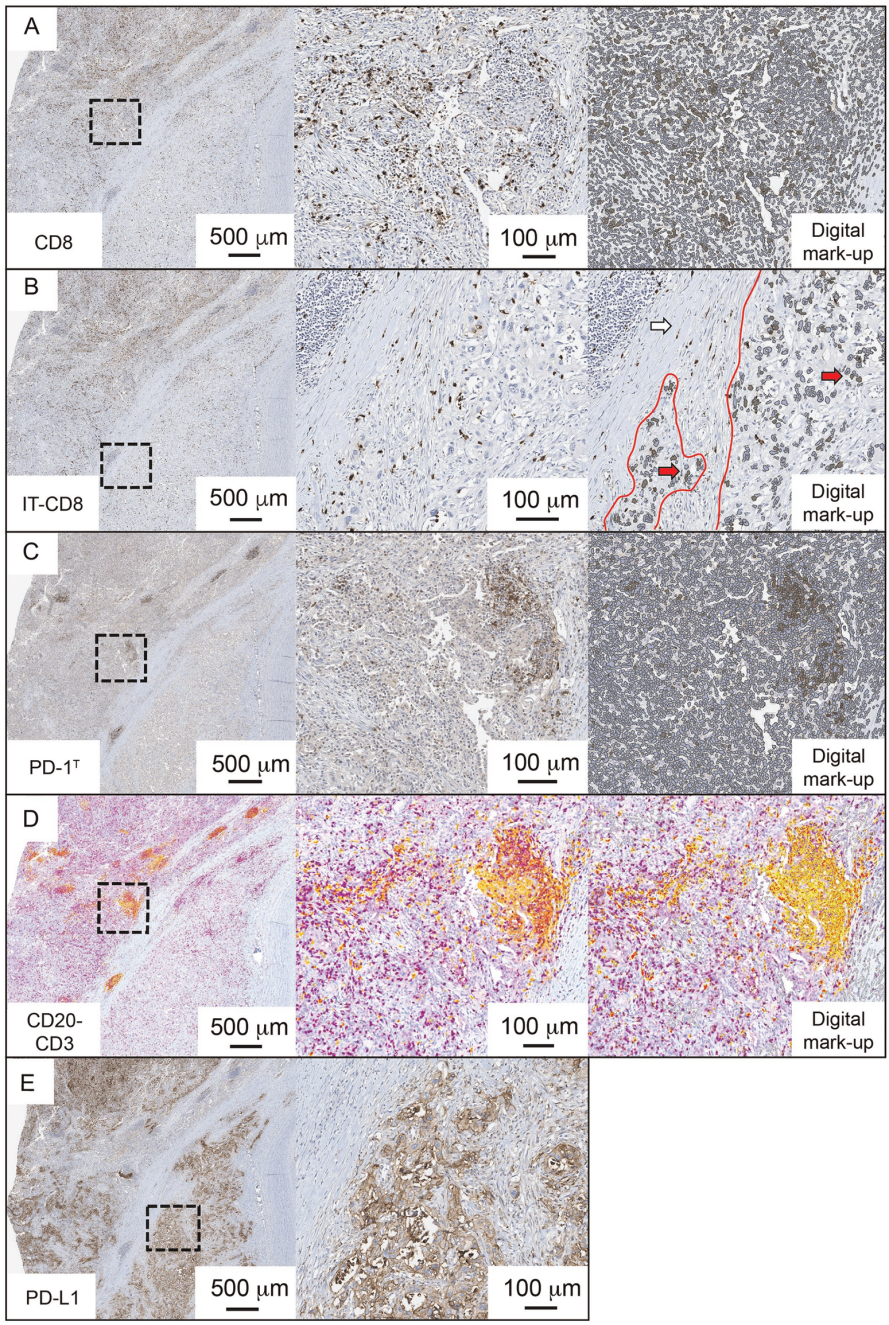
Immunohistochemical analysis of all biomarkers and digital mark-up. **(A)** The left image shows an example of a CD8 immunohistochemical staining (IHC). The black square indicates the area that is shown in the central image. The right image shows the digital markup with CD8 TILs (in brown) and all other cells (in grey). **(B)** The left image shows the same example as shown in A. The black square indicates the area that is shown in the central image. The right image shows regional analysis of only intratumoral (IT) CD8 TILs. Stromal CD8 TILs are not quantified. Red lines indicate the tumor region. Red arrows indicate IT-CD8 TILs. White arrow indicates the area with stromal CD8 TILs. **(C)** The left image shows an example of a consecutive slide stained for PD-1 IHC. The black square indicates the area that is shown in the central image. The right image shows the digital markup with PD-1^T^ TILs (in brown) and all other cells (in grey). **(D)** The left image shows an example of a consecutive slide double stained with CD20 and CD3 IHC. The black square indicates the area that is shown in the central image with CD20^+^ B cells (in yellow) and CD3^+^ T cells (in purple) localizing in a TLS. The right image shows the digital markup with CD20-positive areas highlighted by the intensity of the yellow staining (depicted as spectrum from yellow to red color). **(E)** Example of a consecutive slide stained for PD-L1 IHC. The black square indicates the area that is shown in the right image. PD-L1 IHC slides were scored manually.

To evaluate the predictive performance of various biomarker combinations, we first analyzed pretreatment tumor samples from 162 advanced stage NSCLC patients treated with nivolumab. Nine biomarkers were assessed: (1) the total number of CD8 TILs per mm^2^, (2) the percentage intra-tumoral (IT) CD8 T cells of total CD8 TILs, (3) the number of PD-1^T^ TILs per mm^2^ (4) the CD3-positive area per mm^2^ to estimate the presence of CD3 TILs (5) the CD20-positive area per mm^2^ to estimate the presence of B cells (6) the number of TLS and (7) the combined number of TLS and LA (referred as TLS+LA) per mm^2^, (8) the PD-L1 Tumor Proportion Score (TPS) and (9) the TIS score (NanoString) (Fig 1). CD8 TILs and IT-CD8 T cells were successfully assessed in 132/162 (81%), PD-1^T^ TILs in 103/162 (64%), CD3 TILs, CD20^+^ B cells, TLS and TLS+LA in 128/162 (79%), PD-L1 TPS in 134/162 (83%) and TIS in 68/162 (42%) samples (Table 1, S1 Fig). The use of solely archival samples led to a subset that lacked sufficient tumor tissue, as these samples had been previously used for analyses in the standard diagnostic routine. Additional exclusion criteria for each biomarker are provided in S1 Fig.

**Table 1:** Total number of samples per biomarker in the training and validation cohort.

Biomarkers	Total samples (n)	Training (n)					Validation (n)				
		DC 6m	PD (within 6m)	*DC 12 m*	*PD (within 12m)*	Total	DC 6m	PD (within 6m)	*DC 12m*	*PD (within 12m)*	Total
CD8 TILs IT-CD8 T cells	132	16	39	*12*	*43*	**55**	25	52	*16*	*61*	**77**
PD-1^T^ TILs	103	12	30	*9*	*33*	**42**	18	43	*14*	*47*	**61**
CD3 TILs CD20^+^ B cells TLS TLS+LA	128	16	37	*12*	*41*	**53**	24	51	*15*	*60*	**75**
PD-L1 TPS	134	16	39	*12*	*43*	**55**	25	54	*16*	*63*	**79**
TIS	68	8	20	*6*	*22*	**28**	12	28	*7*	*33*	**40**

Patients with results for at least two biomarkers (n = 135) were randomly assigned to a training (n = 55) and validation (n = 80) cohort. This randomization was stratified for clinical benefit to ensure that in both cohorts, 1 in 3 patients reached disease control at 6 months (DC 6m) and 1 in 5 patients reached disease control at 12 months (DC 12m), respectively. Due to the limited availability of patients with TIS scores (n = 68), these patients were proportionately distributed in the randomization process (Table 1, S1 Fig). Individual results for each biomarker per patient are detailed in S1 Table. No significant differences in demographic characteristics were observed between the training and validation cohorts (Table 2).

**Table 2:** Patient characteristics and treatment outcomes for training and validation cohorts.

	*p-value*	Training cohort	Validation cohort
		n = 55	n = 80
Sex	1.00		
Male, no.(%)		30 (55%)	44 (55%)
Female, no.(%)		25 (45%)	36 (45%)
Age (years), mean (SD^a^)	0.20	62 (10.1)	65 (7.5)
Smoking (never/ex/current)	0.64	5/44/6	12/51/17
Pack years, median (IQR^b^)	0.90	29 (20)	30 (28)
PS^c^, no. (%)	0.46		
0		16 (29%)	16 (20%)
1		29 (53%)	50 (62%)
≥2		10 (18%)	14 (18%)
Pathology, no.(%)	0.19		
Adeno		35 (64%)	50 (62%)
Squamous		10 (18%)	20 (25%)
LCNEC^d^, NSCLC^e^-type		0 (0%)	3 (4%)
NSCLC, NOS^f^		10 (18%)	7 (9%)
Mutations, no. (%)	0.86		
*KRAS*^g^ positive		19 (35%)	30 (38%)
PD-L1^h^ TPS^i^, no. (%)			
Negative <1%	1.00	30 (55%)	43 (54%)
Positive ≥1%		25 (45%)	36 (45%)
Negative <50%	0.66	43 (78%)	65 (81%)
Positive >50%		12 (22%)	14 (18%)
Unknown		0 (0%)	1 (1%)
Brain metastases, no. (%)	0.67	13 (24%)	16 (20%)
Line of treatment, no (%)	0.63		
1		0 (0%)	1 (1%)
2		42 (76%)	56 (70%)
>2		13 (24%)	23 (29%)
Best Overall Response	0.62		
CR^j^/PR^k^		11 (20%)	15 (19%)
SD^l^		5 (9%)	16 (20%)
*SD (PFS <6 months)*		0 (0%)	6 (7%)
*SD (PFS ≥6 months)*		5 (9%)	10 (13%)
PD^m^		39 (71%)	49 (61%)
DC^n^			
at 6 months	0.85	16 (29%)	25 (31%)
at 12 months	0.83	12 (22%)	16 (20%)

*P*-values were calculated by Mann-Whitney, Fisher exact or linear-by-linear association tests.

^a^standard deviation

^b^interquartile range

^c^performance score, based on the European Cooperative Oncology group (ECOG) performance status score. This is a score ranging from 0 to 5, where 0 indicates no symptoms, 1 indicates mild symptoms and above 1 indicates greater disability

^d^large cell neuroendocrine carcinoma

^e^non-small cell lung cance

^f^not otherwise specified

^g^Kirsten Rat Sarcoma viral oncogene

^h^programmed death ligand 1

^i^tumor proportion score

^j^complete response

^k^partial response

^l^stable disease

^m^progressive disease

^n^disease control.

### Accuracy of individual and composite biomarkers to predict DC at 6 months

Next, optimal cut-off values for individual and composite biomarkers in the training cohort were determined through ROC analysis. We aimed for a sensitivity and NPV of ≥90% to minimize the risk of undertreatment, while maintaining a specificity of at least 50% to identify patients unlikely to respond to PD-1 blockade therapy. The latter group can potentially benefit from alternative treatments. Since not all tumor samples were evaluable for all nine biomarkers, the number of samples in the training cohort ranged from 28 to 55 (Table 1, S1 Fig). A total of 16 composite biomarkers, including PD-1^T^ and TIS as individual biomarkers, met the prespecified sensitivity and specificity criteria on the ROC curve (S2 Table). Interestingly, among these, cut-off values of 7/8 (88%) possible combinations with PD-1^T^ TILs and 5/8 (63%) with TIS reached these criteria (S2 Table). However, none significantly improved predictive accuracy compared to the standalone use of PD-1^T^ TILs and TIS (S2A and S2B Fig), leading to their exclusion from further analysis.

**Fig 2:**
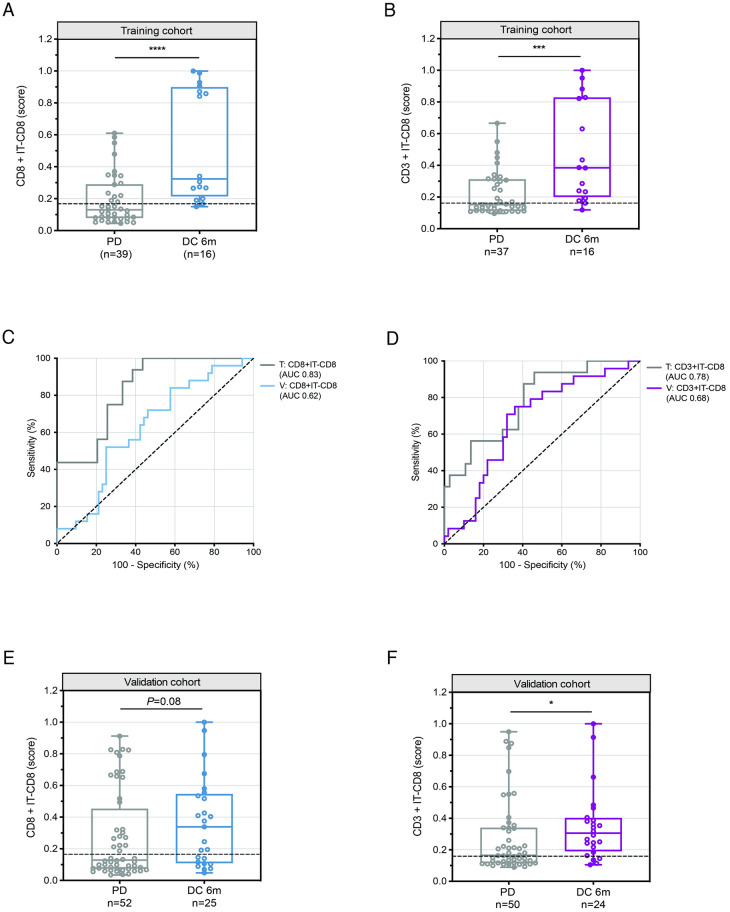
Performance of selected composite and individual biomarkers to predict DC at 6 months in NSCLC patients treated with PD-1 blockade. **(A)** Probability scores of CD8+IT-CD8 in pretreatment samples from patients with disease control at 6 months (DC 6m) (n = 16) and progressive disease (PD) (n = 39) in the training cohort (n = 55). Dashed line indicates a cut-off of 0.167. Medians, interquartile ranges and minimum/maximum shown in boxplots, ****P<0.0001 by Mann Whitney U-test. **(B)** Probability scores of CD3+IT-CD8 in pretreatment samples from patients with DC 6m (n = 16) and PD (n = 37) in the training cohort (n = 53). Dashed line indicates a cut-off of 0.161. Medians, interquartile ranges and minimum/maximum shown in boxplots, ***P<0.001 by Mann Whitney U-test. **(C)** Receiver operating characteristic (ROC) curve for predictive value of CD8+IT-CD8 for DC 6m in the training (n = 55) (AUC 0.83, 95% CI: 0.73–0.94) and validation cohort (n = 77) (AUC 0.62, 95% CI: 0.50–0.75). **(D)** ROC curve for predictive value of CD3+IT-CD8 for DC 6m in the training (n = 53) (AUC 0.78, 95% CI: 0.65–0.91) and validation cohort (n = 74) (AUC 0.68, 95% CI: 0.55–0.80). **(E)** Same plot as in **A** (CD8+IT-CD8) for patients with DC 6m (n = 25) and PD (n = 52) in the validation cohort (n = 77), *P* = 0.08. **(F)** Same plot as in **B** (CD3+IT-CD8) for patients with DC 6m (n = 24) and PD (n = 50) in the validation cohort (n = 74), *P = 0.02.

Subsequently, we selected the four remaining biomarkers with the highest predictive performance for validation, including the combinations of CD8+IT-CD8 and CD3+IT-CD8, as well as the individual biomarkers PD-1^T^ TILs and TIS (S2 Table). In the training cohort, both CD8+IT-CD8 and CD3+IT-CD8 demonstrated significantly higher probability scores in the DC 6m group (reflecting the probability of patients reaching DC 6m) compared to the progressive disease (PD) group (CD8+IT-CD8, *P*<0.0001 and CD3+IT-CD8, *P*<0.001) (Fig 2A and 2B). The area under the ROC curve (AUC) was 0.83 (95% CI 0.73–0.94) for CD8+IT-CD8, and 0.78 (95% CI 0.65–0.92) for CD3+IT-CD8 (Fig 2C and 2D). Corresponding cut-off values of 0.167 and 0.161, respectively, were associated with a sensitivity of 94% for both, specificity of 62% and 54%, NPV of 96% and 95% and PPV of 50% and 47% (Table 3).

**Table 3:** Predictive accuracy of selected individual and composite biomarkers, summary of training and validation results.

					Training					Validation					
Clinical outcome	Bio-marker	Predictor	Cut-off	Samples (n)	*AUC* *(95% CI)*	Sensitivity	Specificity	NPV	PPV	Samples (n)	*AUC (95%-CI)*	Sensitivity	Specificity	NPV	PPV
***DC 6 months***	PD-1^T^ TILs		90	*42*	0.82 (0.69–0.95)	92%	67%	95%	52%	*61*	0.72 (0.57–0.87)	72%	74%	86%	54%
	TIS		6.65	*28*	0.81 (0.65–0.98)	100%	55%	100%	47%	*40*	0.57 (0.36–0.77)	83%	39%	84%	37%
	CD8+ IT-CD-8	$Probability\;for\;DC=\frac{1}{\begin{array}{c} 1-\text{exp}(-3.5749+0.0031*\\ \text{CD}8+0.043*\text{IT}-\text{CD}8) \end{array}}$	0.167	*55*	0.83 (0.73–0.94)	94%	62%	96%	50%	*77*	0.62 (0.50–0.75)	64%	56%	76%	41%
	CD3+ IT-CD-8	$Probability\;for\;DC=\frac{1}{\begin{array}{c} 1-\text{exp}(-2.3821+0.0806*\\ \text{CD}3+0.0175*\text{IT}-\text{CD}8+\\ 0.0069*\text{CD}3*\text{IT}-\text{CD}8) \end{array}}$	0.161	*53*	0.78 (0.65–0.91)	94%	54%	95%	47%	*74*	0.68 (0.55–0.80)	83%	46%	85%	43%
***DC 12 months***	PD-1^T^ TILs		90	*42*	0.82 (0.70–0.94)	100%	64%	100%	43%	*61*	0.80 (0.65–0.94)	86%	74%	95%	50%
	TIS		6.65	*28*	0.77 (0.58–0.96)	100%	50%	100%	35%	*40*	0.63 (0.43–0.82)	100%	39%	100%	26%
	CD8+ IT-CD-8	$Probability\;for\;DC=\frac{1}{\begin{array}{c} 1-\text{exp}(-4.0644+0.003*\\ CD8+0.0436*IT-CD8) \end{array}}$	0.122	*55*	0.85 (0.73–0.96)	92%	63%	96%	41%	*77*	0.67 (0.53–0.81)	68%	57%	88%	30%
	CD8+ TIS	$Probability\;for\;DC=\frac{1}{\begin{array}{c} 1-\text{exp}(-5.7952+0.0224*\\ \text{CD}8+0.2346*\text{TIS}+\\ -0.0021*\text{CD}8*\text{TIS}) \end{array}}$	0.124	*28*	0.91 (0.79–1.00)	100%	68%	100%	46%	*38*	0.59 (0.36–0.82)	29%	68%	81%	17%

Furthermore, PD-1^T^ TIL numbers and TIS scores were significantly higher in the DC 6m group compared to the PD group (PD-1^T^ TILs, *P*<0.001 and TIS, *P*<0.01) (S2C and S2D Fig). PD-1^T^ TILs showed an AUC of 0.82 (95% CI 0.69–0.95), and TIS demonstrated an AUC of 0.81 (95% CI 0.65–0.98) (S2E and S2F Fig). For PD-1^T^ TILs, a cut-off value of 90 per mm^2^ was selected based on its predictive value in a previous study [25]. This threshold yielded a sensitivity of 92%, specificity of 67%, NPV of 95% and PPV of 52% (Table 3). A score of 6.65 was identified as the optimal cut-off for TIS, resulting in a sensitivity of 100%, specificity of 55%, NPV of 100% and PPV of 47% (Table 3).

Next, we evaluated the predictive performance of the four selected biomarkers (CD8+IT-CD8, CD3+IT-CD8, PD-1^T^ TILs and TIS) in the validation cohort. The number of samples with successfully obtained biomarker results in the validation cohort ranged from 40 to 79 (Table 1, S1 Fig). A decrease in the predictive accuracy of CD8+IT-CD8 and CD3+IT-CD8 biomarkers was observed when compared to the training cohort, as reflected by the AUC of the ROC curve measuring 0.62 (95% CI 0.50–0.75) and 0.68 (95% CI 0.55–0.80), respectively (Fig 2C and 2D). Moreover, the probability scores within the DC 6m group did not significantly differ from those in the PD group for CD8+IT-CD8 (*P* = 0.08) (Fig 2E). This comparison for CD3+IT-CD8 was borderline significant (*P* = 0.01) (Fig 2F). The selected cut-off value of CD8+IT-CD8 reached a sensitivity of 64%, specificity of 56%, NPV of 76% and PPV of 41%. The predictive accuracy of CD3+IT-CD8, while higher than that of CD8+IT-CD8, remained lower than in the training cohort (sensitivity: 83%, specificity: 46%, NPV: 85% and PPV: 43%) (Table 3).

The individual biomarker analysis in the validation cohort showed significantly higher PD-1^T^ TIL numbers in the DC 6m group versus the PD group (*P*<0.01), wheras no significant difference was observed for TIS scores (*P* = 0.52) (S2G and S2H Fig). Although the discriminatory ability of PD-1^T^ TILs was lower compared to the training, it still reached an AUC of 0.72 (95% CI 0.57–0.87) (S2E Fig). A cut-off value of 90 PD-1^T^ TILs per mm^2^ resulted in a sensitivity of 72%, specificity of 74%, NPV of 86% and PPV of 54%. Conversely, TIS obtained a low AUC of 0.57 (95% CI 0.36–0.77) (S2F Fig). A TIS score of 6.65 showed a comparable sensitivity (83%), NPV (84%) and PPV (37%) but lower specificity (39%) (Table 3). In summary, these results demonstrate that the combination of CD8+IT-CD8 and CD3+IT-CD8 did not improve predictive accuracy compared to the standalone use of PD-1^T^ TILs and TIS. Furthermore, none of the selected biomarkers met the prespecified performance criteria.

### Accuracy of individual and composite biomarkers to predict DC at 12 months

Approximately 70–80% of patients undergoing second-line treatment with PD-1/PD-L1 blockade experience disease progression within 12 months [2–4]. Our previous work demonstrated the superior efficacy of PD-1^T^ TILs in identifying patients with DC at 12 months (DC 12m) compared to those with DC 6m, as well as identifying a subgroup without long-term benefit [25]. In light of this, we extended our analysis to evaluate the predictive performance of all biomarkers for DC 12m. Similar to the analysis performed for DC 6m, we constructed ROC curves to determine optimal cut-off values corresponding to a sensitivity of ≥90% and specificity of ≥50% for each composite and individual biomarker. Four patients in the training and nine patients in the validation cohort experienced disease progression between 6 and 12 months, allocating them into the PD group. In the training cohort, 16 composite biomarkers, along with individual biomarkers PD-1^T^ TILs and TIS, met the prespecified sensitivity and specificity ciritera (S3 Table). PD-1^T^ TIL and TIS combinations did not significantly improve predictive accuracy (S3A and S3B Fig, S3 Table). Noteworthy, the combination of CD8 with TIS (CD8+TIS) showed an 18% increase in specificity compared to TIS alone. Despite not reaching statistical significance, possibly due to the low sample size (*P* = 0.34), this combination was selected for further analysis (S3B Fig, S3 Table). Other selected biomarkers for validation with the highest predictive performance included CD8+IT-CD8, PD-1^T^ TILs and TIS (S3 Table).

**Fig 3:**
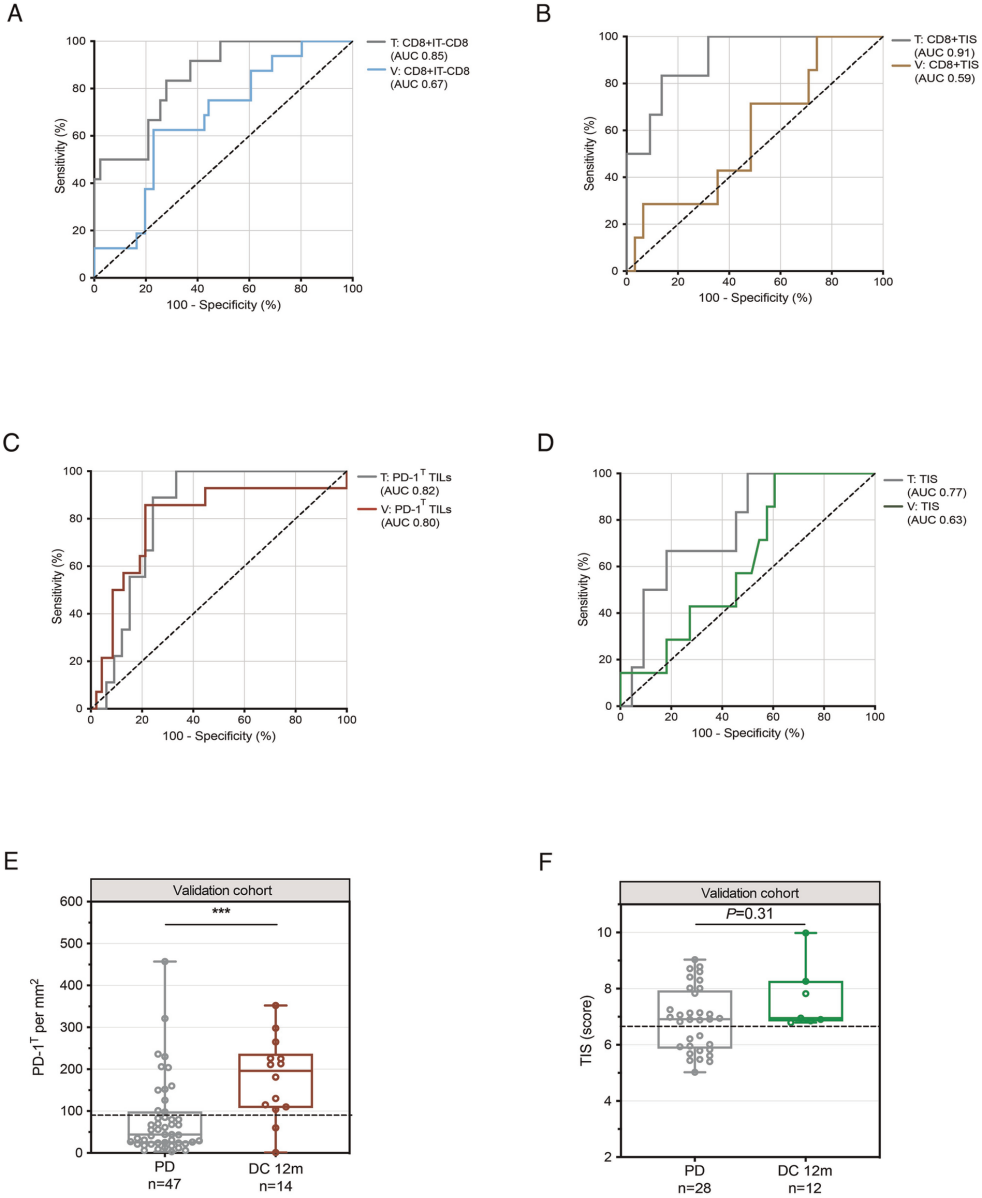
Performance of selected composite and individual biomarkers to predict DC at 12 months in NSCLC patients treated with PD-1 blockade. **(A)** Receiver operating characteristic (ROC) curve for predictive value of CD8+IT-CD8 for disease control at 12 months (DC 12m) in the training cohort (n = 55) (AUC 0.85, 95% CI: 0.73–0.96) and validation cohort (n = 77) (AUC 0.67, 95% CI: 0.53–0.81). **(B)** ROC curve for predictive value of CD8+TIS for DC 12m in the training cohort (n = 28) (AUC 0.91, 95% CI: 0.79–1.00) and validation cohort (n = 38) (AUC 0.59, 95% CI: 0.36–0.82). **(C)** ROC curve for predictive value of PD-1^T^ TILs for DC 12m in the training cohort (n = 42) (AUC 0.82, 95% CI: 0.70–0.94) and validation cohort (n = 61) (AUC 0.80, 95% CI: 0.65–0.94). **(D)** ROC curve for predictive value of TIS for DC 12m in the training cohort (n = 28) (AUC 0.77, 95% CI: 0.58–0.96) and validation cohort (n = 40) (AUC 0.63, 95% CI: 0.43–0.82). **(E)** PD-1^T^ TILs per mm^2^ in pretreatment samples from patients with DC 12m (n = 14) and PD (n = 47) in the validation cohort (n = 61). Dashed line indicates a cut-off of 90 PD1^T^ TILs per mm^2^. Medians, interquartile ranges and minimum/maximum shown in boxplots, **P<0.01 by Mann Whitney U-test. **(F)** TIS scores in pretreatment samples from patients with DC 12m (n = 7) and PD (n = 33) in the validation cohort (n = 40). Dashed line indicates a cut-off score of 6.65. Medians, interquartile ranges and minimum/maximum shown in boxplots, *P* = 0.31 by Mann Whitney U-test.

The probability scores for DC 12m and PD are depicted per sample in S3C Fig (CD8+IT-CD8, *P*<0.001) and S3D Fig (CD8+TIS, *P*<0.01). The two composite biomarkers showed a high AUC of 0.85 (95% CI: 0.73–0.96) (CD8+IT-CD8) and 0.91 (95% CI: 0.79–1.00) (CD8+TIS) in the training cohort, and optimal cut-off values of 0.122 and 0.124, respectively, were chosen (Fig 3A and 3B, Table 3). In the validation cohort, only CD8+IT-CD8 showed borderline significantly higher probability scores in the DC 12m group versus the PD group (*P* = 0.03) (S3E and S3F Fig). The ROCs resulted in low AUCs (CD8+IT-CD8: 0.67 (95% CI: 0.53–0.81), CD8+TIS: 0.59 (95% CI 0.36–0.81)) (Fig 3A and 3B). Furthermore, the sensitivity (68% and 29%), specificity (57% and 68%), NPV (88% and 81%) and PPV (30% and 17%) did not meet the prespecified performance criteria (Table 3).

PD-1^T^ TIL numbers and TIS scores for the training cohort are presented in S3G and S3H Fig. A cut-off value of 90 PD-1^T^ TILs per mm^2^ and a TIS score of 6.65 demonstrated similar predictive accuracy as observed in the training cohort for DC 6m (Fig 3C and 3D, Table 3). In the validation cohort, PD-1^T^ TIL numbers were significantly higher in patients with DC 12m versus PD (*P*<0.001) (Fig 3E). PD-1^T^ TILs maintained a consistently high AUC of 0.80 (95% CI: 0.65–0.94) and demonstrated good performance (sensitivity: 86%, specificity: 74%, NPV: 95% and PPV: 50%) (Fig 3C, Table 3). A subgroup analysis revealed an enrichment of patients with DC 12m in the PD-1^T^ TILs ≥90 group and patients with PD in the PD-1^T^ TILs <90 subgroup (Fig 3E). TIS scores did not significantly differ between the two groups (*P* = 0.31) and showed a low AUC of 0.63 (95% CI 0.43–0.82) (Fig 3D and 3F). Nevertheless, a cut-off score of 6.65 reached a sensitivity of 100%, specificity of 39%, NPV of 100% and PPV of 26% (Table 3). Although these findings did not meet the prespecified ≥50% specificity criterium, they accurately identified all patients with DC 12m including 39% of patients with PD. Taken together, PD-1^T^ TILs and TIS, as individual biomarkers, showed superior predictive accuracy for DC 12m compared to CD8+IT-CD8 and CD8+TIS. Notably, PD-1^T^ TILs alone demonstrated greater performance than TIS alone, particularly in terms of specificity and PPV.

## Discussion

Since the introduction of PD-1/PD-L1 blockade therapy, the clinical outcomes for advanced stage NSCLC have markedly improved. Nevertheless, a minor subset of patients derive benefit from these treatments, leading to concerns of overtreatment and unnecessary side effects. In addition, healthcare systems deal with increasing costs. Several predictive biomarkers have been identified to support treatment decision-making. Given the complex interactions within the TME influencing the immune response during PD-1/PD-L1 blockade therapy, the likelihood of identifying a single perfect biomarker is minimal. Here, we evaluate the predictive performance of combinations of biomarkers in a cohort of advanced-stage NSCLC patients treated with nivolumab. Our findings show that the selected composite biomarkers did not improve predictive performance compared to PD-1^T^ TILs and TIS alone. At 6 months, none of the selected biomarkers met the prespecified criteria of ≥90% sensitivity and ≥50% specificity in the validation cohort. However, at 12 months, PD-1^T^ TILs and TIS demonstrated a high sensitivity in identifying patients with DC 12m with. Patients without long-term benefit were more accurately identified by PD-1^T^ TILs than TIS.

While CD8 or CD3 TILs, in combination with intratumoral localization of CD8 T cells, emerged as the most accurate composite biomarkers for DC 6m in the training cohort, their discriminatory ability was notably low in the validation cohort. The mere presence and localization of TILs may not indicate that all T cells are in a state to recognize and eliminate the tumor [37, 38]. In the present study, this notion is supported by the high predictive accuracy of PD-1^T^ TILs for DC 12m, given that these TILs constitute a distinct subset with an enhanced capacity for tumor recognition [24]. The consistency of these findings with our previous work can be attributed to the predominant reuse of samples [25]. Further refinement of this specific T cell population holds promise for the development of new markers or gene signatures, as shown by various studies [39–41]. Moreover, we recently developed a clinically applicable mRNA expression signature reflecting the presence of PD-1^T^ TILs in the TME [35]. Since most biomarkers assessed in this study are associated wit antitumor immunity and are presumably correlated, PD-1^T^ combinations did not improve specificity.

Previous studies have shown the predictive potential of combining CD8+PD-L1 [22, 34]. However, in the training cohort, the CD8+PD-L1 combination failed to meet the sensitivity and specificity criteria, leading to its exclusion from further evaluation. PD-L1 expression on tumor cells is transient and relies on the production of IFNγ by TILs [42]. This dynamic nature of PD-L1 expression could contribute to variable tumor PD-L1 expresssion in the training samples, potentially impacting the predictive accuracy of PD-L1 and of PD-L1 combinations. Furthermore, it is important to note that this study is limited by the number of samples, particularly for TIS assessment. Therefore, we restricted our evaluation to two-biomarker combinations instead of considering three or more. Studies with a larger sample size are essential to validate the robustness of our findings.

Our results for TIS align with other studies that have demonstrated the predictive potential of this signature [29, 43]. Interestingly, TIS includes genes, such as *LAG3* and *TIGIT*, that are highly expressed in PD-1^T^ TILs [24, 29]. A high number of PD-1^T^ TILs or a high TIS score in pretreatment samples may serve as surrogate markers for a tumor’s capacity to undergo durable immune reactivation upon PD-1 blockade therapy. A PD-1^T^ TILs or TIS combination with biomarkers representing distinct facets of the immune response holds promise for improving predictive accuracy. For instance, TMB can serve as a read-out for immunogenic neoantigens arising from somatic mutations [15]. Previous studies have identified TMB and PD-L1 as independent predictors for advanced NSCLC treated with PD-1 blockade and have shown improved performance when combined [32, 33]. Conversely, the presence of tumor-resident regulatory T cells (T_reg_) in the TME might be considered. T_reg_ cells, known for their immune-inhibitory functions, are associated with poor patient survival when present in high numbers [44]. A combination of TMB or T_reg_ with either PD-1^T^ TILs or TIS warrants further investigation in future studies.

In conclusion, this study showed that the biomarker combinations assessed here did not improve predictive performance compared to PD-1^T^ TILs and TIS alone. PD-1^T^ TILs showed the highest predictive performance of all the biomarkers, accurately identifying patients without long-term benefit with high specificity and NPV.

## Supporting information

S1 Fig Flow chart of sample numbers and exclusion criteria per biomarker. In 27 samples none of the biomarkers were assessed. The right grey boxes indicate the exclusion criteria per biomarker and the right light grey boxes indicate the total number of samples that were assessed per biomarker. The remaining samples with ≥2 biomarker values were randomized in a training (n = 55) and validation cohort (n = 80). The blue boxes indicate the number of samples that were assessed per biomarker in the training (T) and validation (V) cohort.
(TIF)

S2 Fig Performance of PD-1^T^ TILs and TIS as individual and as composite biomarker to predict disease control at 6 months (DC 6m) in NSCLC patients treated with PD-1 blockade. (A) Specificity correlating to a sensitivity of ≥90% for combinations with PD-1^T^ TILs as predictive biomarker for DC 6m in the training cohort (n = 27 or n = 42). The grey dashed line indicates the prespecified specificity criterium of ≥50%. Different composite biomarkers were compared to the predictive performance of PD-1^T^ TILs alone. *P* values were calculated by McNemar test. (B) Same plot as in A for combinations with TIS in the training cohort (n = 27 or n = 28). (C) PD-1^T^ TILs per mm^2^ in pretreatment samples from patients with DC 6m (n = 12) and progressive disease (PD) (n = 30) in the training cohort (n = 42). Dashed line indicates a cut-off of 90 PD1^T^ TILs per mm^2^. Medians, interquartile ranges and minimum/maximum shown in boxplots, ***P<0.001 by Mann Whitney U-test. (D) TIS scores in pretreatment samples from patients with DC 6m (n = 8) and PD (n = 20) in the training cohort (n = 28). Dashed line indicates a cut-off score of 6.65. Medians, interquartile ranges and minimum/maximum shown in boxplots, **P<0.01 by Mann Whitney U-test. (E) Receiver operating characteristic (ROC) curve for predictive value of PD-1^T^ TILs for DC 6m in the training cohort (n = 42) (AUC 0.82, 95% CI: 0.69–0.95) and validation cohort (n = 61) (AUC 0.72, 95% CI: 0.57–0.87). (F) ROC curve for predictive value of TIS for DC 6m in the training cohort (n = 28) (AUC 0.81, 95% CI: 0.65–0.98) and validation cohort (n = 40) (AUC 0.57, 95% CI: 0.36–0.77) (G) Same plot as in C (PD-1^T^ TILs) for patients with DC 6m (n = 18) and PD (n = 43) in the validation cohort (n = 61), **P<0.01 by Mann Whitney U-test. (H) Same plot as in D (TIS) for patients with DC 6m (n = 12) and PD (n = 28), *P* = 0.52 by Mann Whitney U-test.
(TIF)

S3 Fig Performance of PD-1^T^ TILs and TIS as individual and as composite biomarker to predict disease control at 12 months (DC 12m) and association of CD8+IT-CD8 and CD8+TIS with DC 12m in NSCLC patients treated with PD-1 blockade. (A) Specificity correlating to a sensitivity and NPV of ≥90% for combinations with PD-1^T^ TILs as predictive biomarker for DC 12m in the training cohort (n = 27 or n = 42). The grey dashed line indicates the prespecified specificity criterium of ≥50%. Different composite biomarkers were compared to the predictive performance of PD-1^T^ TILs alone. *P* values were calculated by McNemar test. (B) Same plot as in A for combinations with TIS in the training cohort (n = 27 or n = 28). (C) Probability scores of CD8+IT-CD8 in pretreatment samples from patients with DC 12m (n = 12) and progressive disease (PD) (n = 43) in the training cohort (n = 55). Dashed line indicates a cut-off of 0.122. Medians, interquartile ranges and minimum/maximum shown in boxplots, ***P<0.001 by Mann Whitney U-test. (D) Probability scores of CD8+TIS in pretreatment samples from patients with DC 12m (n = 6) and PD (n = 22) in the training cohort (n = 28). Dashed line indicates a cut-off of 0.124. Medians, interquartile ranges and minimum/maximum shown in boxplots, **P<0.01 by Mann Whitney U-test. (E) Probability scores of CD8+IT-CD8 in pretreatment samples from patients with DC 12m (n = 16) and PD (n = 61) in the validation cohort (n = 77). Dashed line indicates a cut-off of 0.122. Medians, interquartile ranges and minimum/maximum shown in boxplots, *P = 0.03 by Mann Whitney U-test. (F) Probability scores of CD8+TIS in pretreatment samples from patients with DC 12m (n = 7) and PD (n = 31) in the validation cohort (n = 38). Dashed line indicates a cut-off of 0.124. Medians, interquartile ranges and minimum/maximum shown in boxplots, *P* = 0.48 by Mann Whitney U-test. (G) PD-1^T^ TILs per mm^2^ in pretreatment samples from patients with DC 12m (n = 9) and PD (n = 33) in the training cohort (n = 42). Dashed line indicates a cut-off of 90 PD1^T^ TILs per mm^2^. Medians, interquartile ranges and minimum/maximum shown in boxplots, **P<0.01 by Mann Whitney U-test. (H) TIS scores in pretreatment samples from patients with DC 12m (n = 6) and PD (n = 22) in the training cohort (n = 28). Dashed line indicates a cut-off score of 6.65. Medians, interquartile ranges and minimum/maximum shown in boxplots, *P = 0.04 by Mann Whitney U-test.
(TIF)

S1 Table Overview of all analyzed biomarkers per patient. (XLSX)

S2 Table Predictive accuracy of individual and composite biomarkers at a prespecified criterium of ≥90% sensitivity for detecting disaese control (DC) at 6 months in the training cohort. * indicates an interaction term.
(XLSX)

S3 Table Predictive accuracy of individual and composite biomarkers at a prespecified criterium of ≥90% sensitivity for detecting disaese control (DC) at 12 months in the training cohort. * indicates an interaction term.
(XLSX)
