# Supplementary figures and images for: Head-to-head comparison of composite and individual biomarkers to predict clinical benefit to PD-1 blockade in non-small cell lung cancer
Source: PLoS One. 2024 Jul 31;19(7):e0293707. doi: 10.1371/journal.pone.0293707 (PMC11290656; doi:10.1371/journal.pone.0293707)

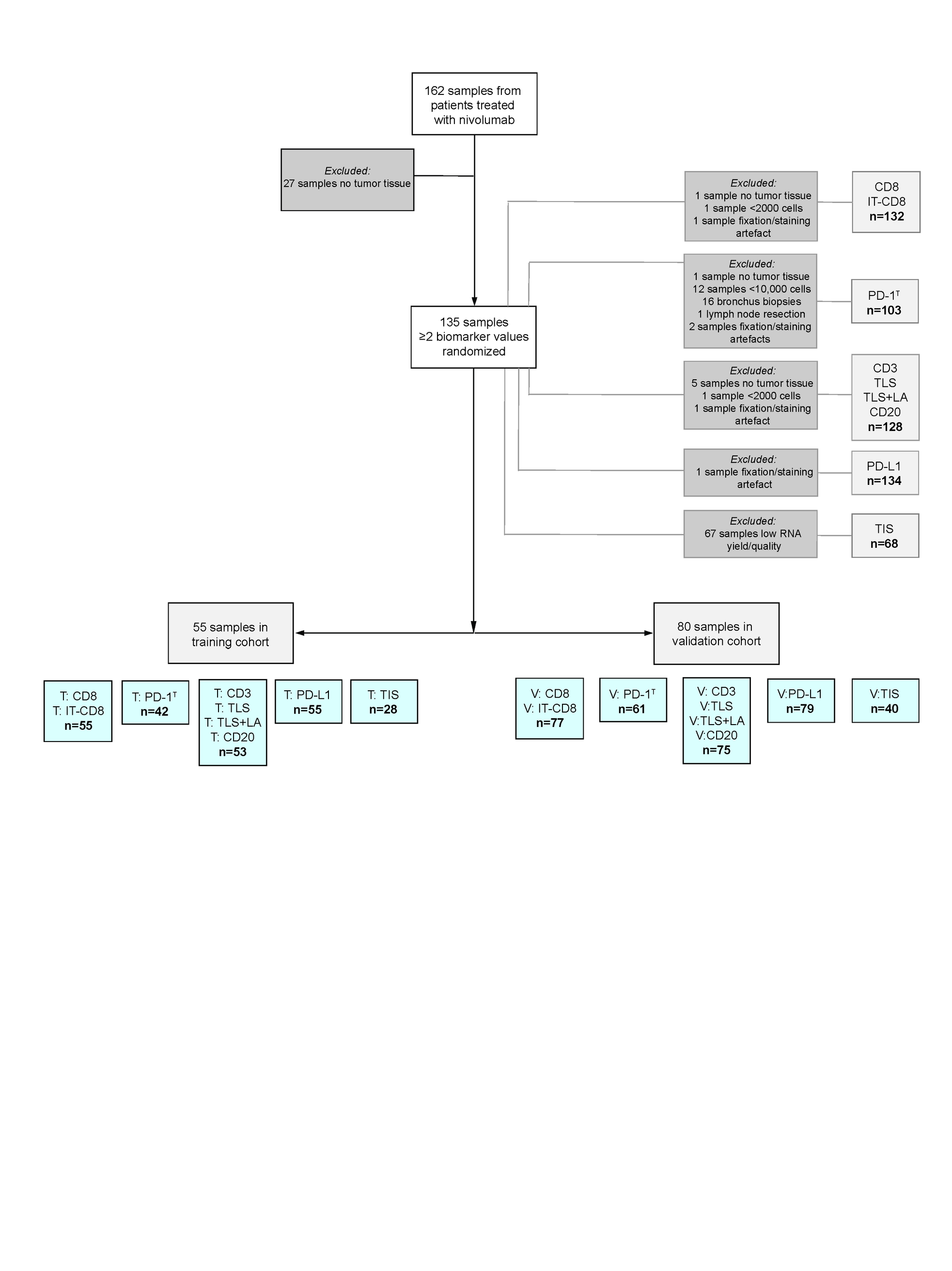

Supplement: S1 Fig — In 27 samples none of the biomarkers were assessed. The right grey boxes indicate the exclusion criteria per biomarker and the right light grey boxes indicate the total number of samples that were assessed per biomarker. The remaining samples with ≥2 biomarker values were randomized in a training (n = 55) and validation cohort (n = 80). The blue boxes indicate the number of samples that were assessed per biomarker in the training (T) and validation (V) cohort. (TIF) [file pone.0293707.s001.tif]

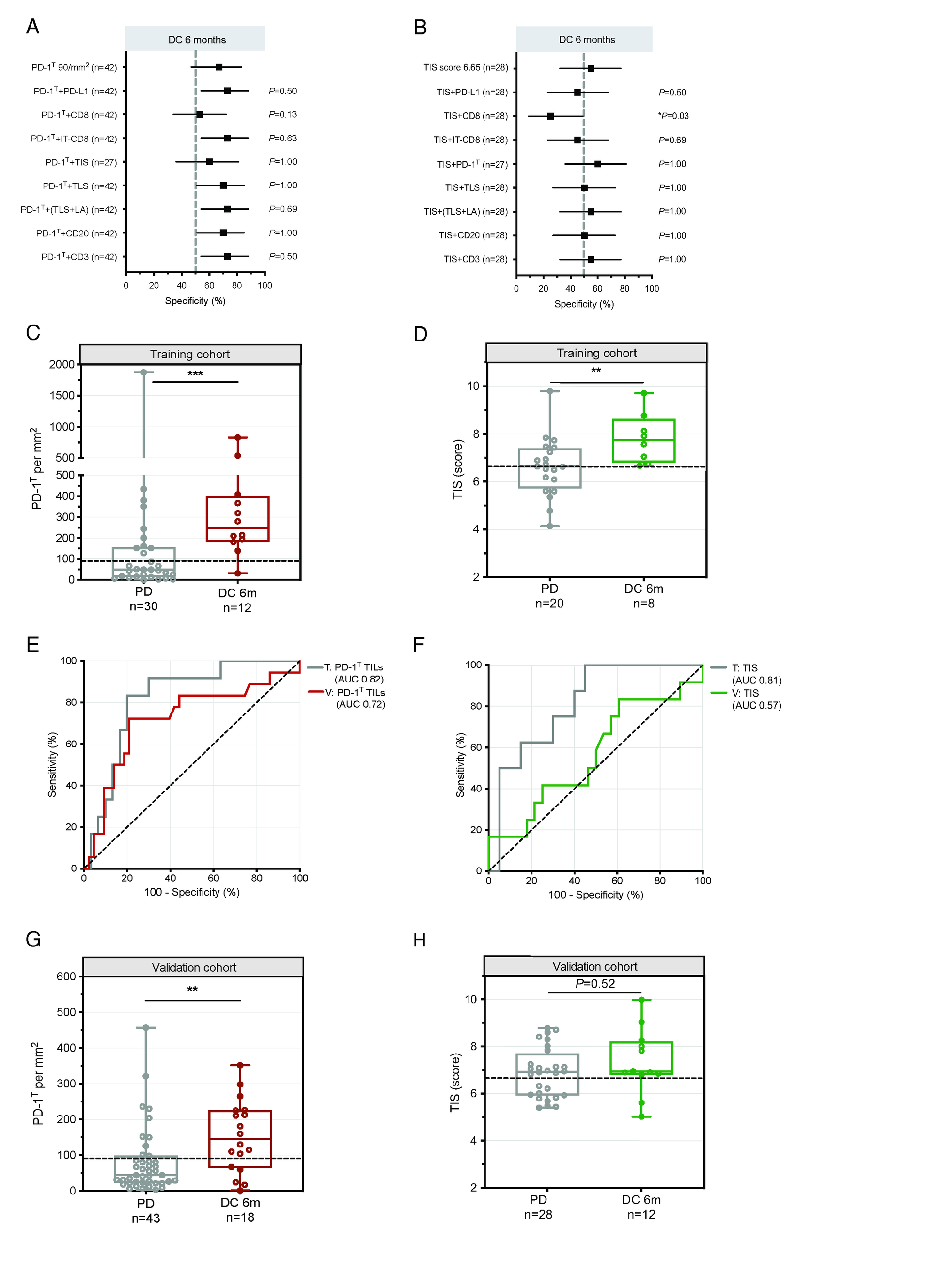

Supplement: S2 Fig — (A) Specificity correlating to a sensitivity of ≥90% for combinations with PD-1T TILs as predictive biomarker for DC 6m in the training cohort (n = 27 or n = 42). The grey dashed line indicates the prespecified specificity criterium of ≥50%. Different composite biomarkers were compared to the predictive performance of PD-1T TILs alone. P values were calculated by McNemar test. (B) Same plot as in A for combinations with TIS in the training cohort (n = 27 or n = 28). (C) PD-1T TILs per mm2 in pretreatment samples from patients with DC 6m (n = 12) and progressive disease (PD) (n = 30) in the training cohort (n = 42). Dashed line indicates a cut-off of 90 PD1T TILs per mm2. Medians, interquartile ranges and minimum/maximum shown in boxplots, ***P<0.001 by Mann Whitney U-test. (D) TIS scores in pretreatment samples from patients with DC 6m (n = 8) and PD (n = 20) in the training cohort (n = 28). Dashed line indicates a cut-off score of 6.65. Medians, interquartile ranges and minimum/maximum shown in boxplots, **P<0.01 by Mann Whitney U-test. (E) Receiver operating characteristic (ROC) curve for predictive value of PD-1T TILs for DC 6m in the training cohort (n = 42) (AUC 0.82, 95% CI: 0.69–0.95) and validation cohort (n = 61) (AUC 0.72, 95% CI: 0.57–0.87). (F) ROC curve for predictive value of TIS for DC 6m in the training cohort (n = 28) (AUC 0.81, 95% CI: 0.65–0.98) and validation cohort (n = 40) (AUC 0.57, 95% CI: 0.36–0.77) (G) Same plot as in C (PD-1T TILs) for patients with DC 6m (n = 18) and PD (n = 43) in the validation cohort (n = 61), **P<0.01 by Mann Whitney U-test. (H) Same plot as in D (TIS) for patients with DC 6m (n = 12) and PD (n = 28), P = 0.52 by Mann Whitney U-test. (TIF) [file pone.0293707.s002.tif]

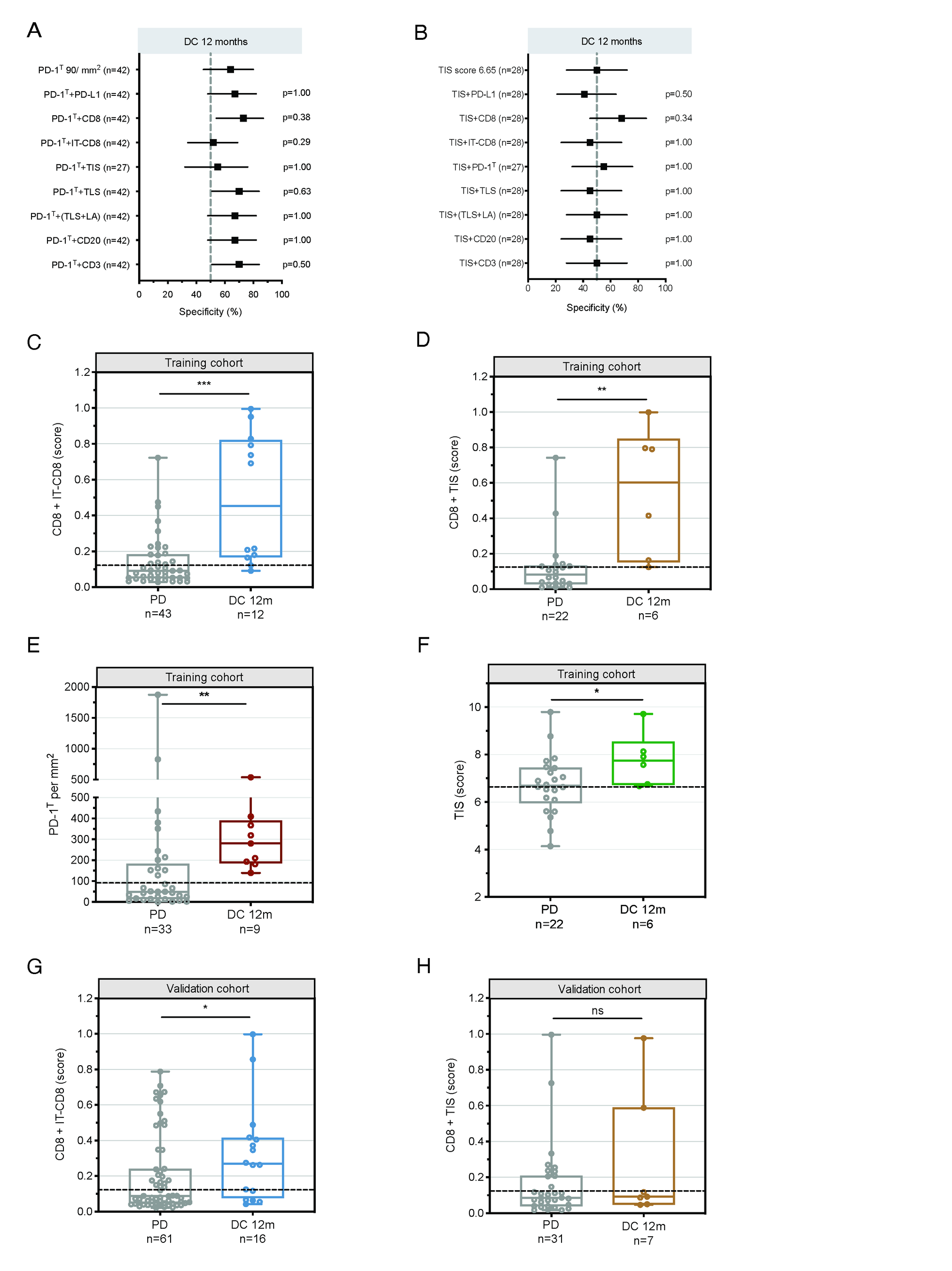

Supplement: S3 Fig — (A) Specificity correlating to a sensitivity and NPV of ≥90% for combinations with PD-1T TILs as predictive biomarker for DC 12m in the training cohort (n = 27 or n = 42). The grey dashed line indicates the prespecified specificity criterium of ≥50%. Different composite biomarkers were compared to the predictive performance of PD-1T TILs alone. P values were calculated by McNemar test. (B) Same plot as in A for combinations with TIS in the training cohort (n = 27 or n = 28). (C) Probability scores of CD8+IT-CD8 in pretreatment samples from patients with DC 12m (n = 12) and progressive disease (PD) (n = 43) in the training cohort (n = 55). Dashed line indicates a cut-off of 0.122. Medians, interquartile ranges and minimum/maximum shown in boxplots, ***P<0.001 by Mann Whitney U-test. (D) Probability scores of CD8+TIS in pretreatment samples from patients with DC 12m (n = 6) and PD (n = 22) in the training cohort (n = 28). Dashed line indicates a cut-off of 0.124. Medians, interquartile ranges and minimum/maximum shown in boxplots, **P<0.01 by Mann Whitney U-test. (E) Probability scores of CD8+IT-CD8 in pretreatment samples from patients with DC 12m (n = 16) and PD (n = 61) in the validation cohort (n = 77). Dashed line indicates a cut-off of 0.122. Medians, interquartile ranges and minimum/maximum shown in boxplots, *P = 0.03 by Mann Whitney U-test. (F) Probability scores of CD8+TIS in pretreatment samples from patients with DC 12m (n = 7) and PD (n = 31) in the validation cohort (n = 38). Dashed line indicates a cut-off of 0.124. Medians, interquartile ranges and minimum/maximum shown in boxplots, P = 0.48 by Mann Whitney U-test. (G) PD-1T TILs per mm2 in pretreatment samples from patients with DC 12m (n = 9) and PD (n = 33) in the training cohort (n = 42). Dashed line indicates a cut-off of 90 PD1T TILs per mm2. Medians, interquartile ranges and minimum/maximum shown in boxplots, **P<0.01 by Mann Whitney U-test. (H) TIS scores in pretreatment samples from patients w [file pone.0293707.s003.tif]
